# Synergistically Enhanced Inhibitory Effects of Pullulan Nanoparticle-Mediated Co-Delivery of Lovastatin and Doxorubicin to Triple-Negative Breast Cancer Cells

**DOI:** 10.1186/s11671-019-3146-0

**Published:** 2019-09-13

**Authors:** Di Wu, Yao Chen, Shun Wen, Yi Wen, Rong Wang, Qiuting Zhang, Ge Qin, Huimei Yi, Mi Wu, Lu Lu, Xiaojun Tao, Xiyun Deng

**Affiliations:** 1 Key Laboratory of Study and Discovery of Small Targeted Molecules of Hunan Province, Hunan Normal University School of Medicine, Changsha, 410013 Hunan China; 2 Key Laboratory of Translational Cancer Stem Cell Research, Department of Basic Medical Sciences, Hunan Normal University School of Medicine, Changsha, 410013 Hunan China

**Keywords:** Lovastatin, Triple-negative breast cancer, Amphiphilic conjugate, Synergistic effect, Nuclear magnetic resonance spectroscopy

## Abstract

Triple-negative breast cancer (TNBC) is a subtype of breast cancer that is prone to drug resistance and difficult to treat. In this study, we grafted water-soluble pullulan with lovastatin (LV) to develop a novel amphiphilic conjugate, pullulan-encapsulated LV (PLV). The PLV conjugate was synthesized with three different ratios of pullulan to LV and characterized by Fourier transform infrared (FTIR). The degree of substitution (DS) of LV in terms of molar ratio was 7.87%, 3.58%, and 3.06% for PLV (1/2), PLV (1/3), and PLV (1/4), respectively, by proton NMR analysis. We selected the PLV (1/2) conjugate to prepare doxorubicin (DXR)-loaded PLV nanoparticles (PLV/DXR NPs) because of its superior properties. The average size and zeta potential for PLV (1/2) NPs were 177.6 nm and − 11.66 mV, respectively, determined by dynamic light scattering, and those for PLV/DXR NPs were 225.6 nm and − 10.51 mV, respectively. In vitro drug release profiling showed that PLV/DXR NPs sustainably released DXR within 72 h, which was more robust at pH 5.4 (97.90%) than pH 7.4 (76.15%). In the cytotoxicity study, PLV/DXR NPs showed greater inhibition of proliferation of TNBC MDA-MB-231 than non-TNBC MDA-MB-453 cells (IC_50_ 0.60 vs 11.05 μM). FITC-loaded PLV/DXR NPs were prepared to investigate cellular uptake: both cell lines showed a time-dependent uptake of NPs, but the number of NPs entering MDA-MB-231 cells was greater than that entering the MDA-MB-453 cells. Pullulan-based NP co-delivery of LV and DXR could efficiently inhibit TNBC cells, which may help in designing a powerful drug delivery system for treating TNBC.

## Introduction

Triple-negative breast cancer (TNBC) is a special subtype of breast cancer that lacks an estrogen receptor (ER), progesterone receptor (PR), and human epidermal growth factor receptor 2 (HER2) and has worse prognosis than other breast cancer subtypes [1–3]. Available targeted therapies mostly depend on targeting specific receptors on the surface of the tumor cell. Because TNBC lacks a specific surface marker for active targeting for treatment, other targeting approaches are worth exploring. For example, passive targeting, which involves a unique enhanced permeability and retention (EPR) effect on solid tumors [4], allows for particles of a specific size range to target the tumor site [5, 6]. A nanoparticle (NP) is a kind of drug carrier that can take advantage of the EPR effect and become enriched in solid tumor sites [4].

Recently, we have shown that lovastatin (LV), one of the lipophilic statins used to treat hyperlipidemia [7], selectively inhibits TNBC by targeting cancer stem cells [8, 9]. Furthermore, we demonstrated that NP encapsulation can enhance the inhibitory effect of LV on TNBC in a mouse model of breast cancer cell implantation [8]. Our studies, together with previous findings [10, 11], suggest that lipophilic statins, particularly LV, can be repurposed as anti-cancer drugs for TNBC treatment. However, whether LV can overcome chemo-resistance or enhance the therapeutic effects of chemotherapeutic drugs in TNBC remains unknown.

The synergy between LV and different types of chemotherapeutic agents such as doxorubicin (DXR), 5-fluorouracil [12], cisplatin [13], and 1-β-D-arabinofuranosylcytosine [14] has been reported in different cancer cell types. DXR is an anthracycline compound with intricate features of binding to DNA and inhibiting nucleic acid synthesis. In addition, LV induced apoptosis of ovarian cancer cells in synergism with DXR [15]. Also, LV is a direct inhibitor of the efflux protein P-glycoprotein [16], an ATP-driven pump that can readily discharge DXR to the extracellular compartment [17, 18]. Thus, LV may increase the therapeutic effect of other therapeutic agents, and the combination of LV and these drugs may enhance the therapeutic efficacy against TNBC.

The current combination therapies are full of challenges. On one hand, combinational chemotherapy may be seriously limited by the fast metabolism, poor water solubility, and low bioavailability of chemotherapeutic drugs [19, 20]. On the other, with the varying pharmacokinetics, membrane transport properties, and non-specific bio-distribution of different drugs, achieving sufficient concentrations and specific ratios of administrated drugs in individual cells is difficult [21–23]. These factors affect the therapeutic efficacy and the level of synergism or antagonism for the combination. Co-encapsulating two therapeutic agents into a nanoscale drug carrier is an efficient way to overcome these challenges because it allows the drugs loaded in the carrier to maintain a correct ratio for the synergistic effects [24, 25].

**Scheme 1:**
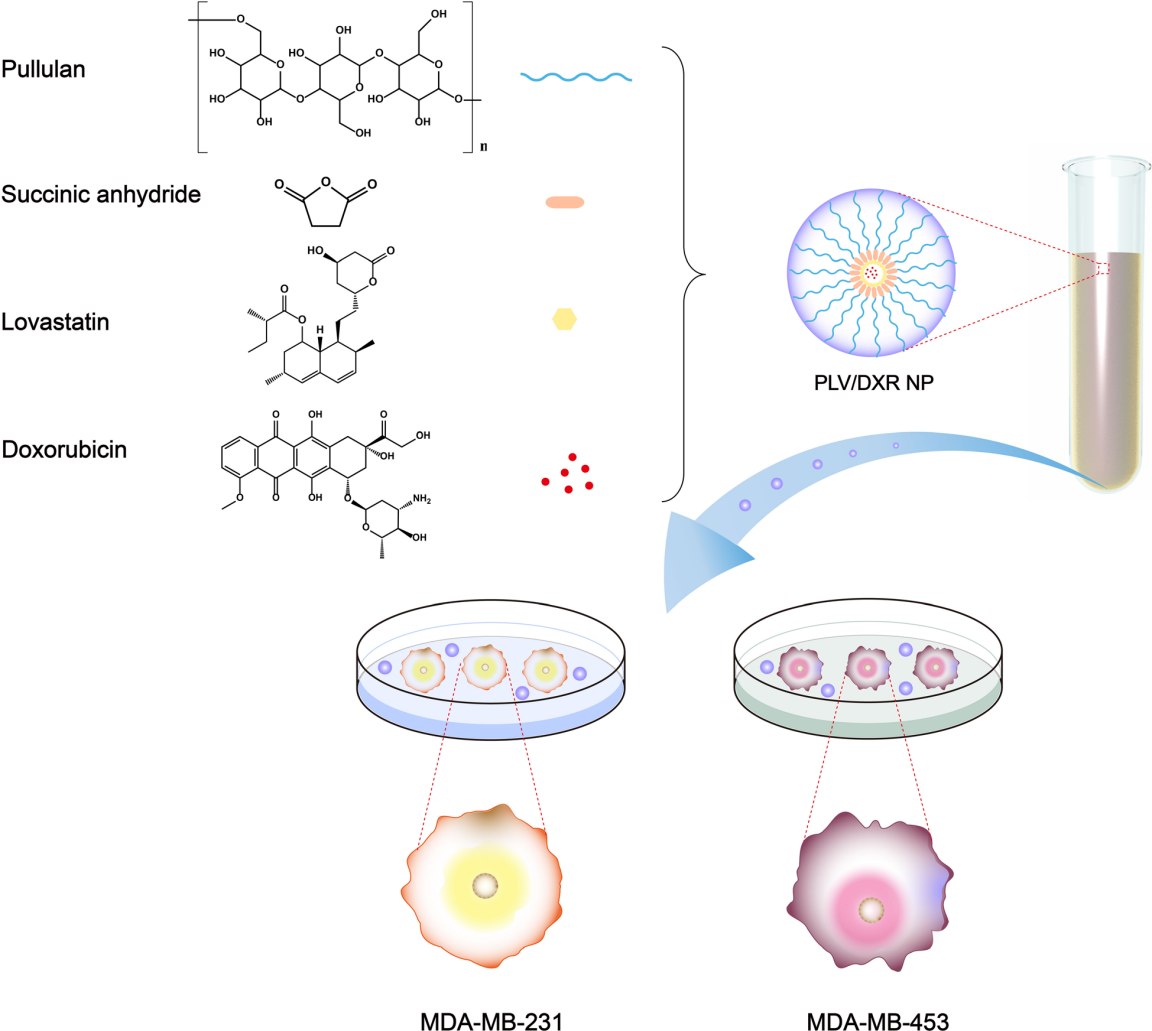
Schematic illustration of nanoparticles (NPs) and in vitro cell experiment. The pullulan-lovastatin (PLV) NP was formed by self-assembly of a polymer formed by pullulan and LV in an aqueous solution. In the process of forming the NP, DXR was loaded in the hydrophobic core of the NP. Then, PLV/DXR was used for in vitro cell experiments with MDA-MB-231 and MDA-MB-453 cells representing triple-negative breast cancer (TNBC) and non-TNBC cells, respectively

Co-encapsulated nanocarriers mainly include liposomes, polymeric micelles, and micro- or nano-spheres [26–29]. Polymeric micelles, because of their superior performance (e.g., with chemically conjugated and physically encapsulated dual drug loading, high drug loading, and high biocompatibility), has attracted much attention [26, 30]. In our previous study, we prepared a series of NPs with pullulan, a hydrophilic polysaccharide with high biocompatibility, and studied the effects of various factors on their drug release behavior [31]. In this current study, we prepared a novel PLV polymeric micelle with different feed ratios of pullulan and LV and characterized the distribution of size and zeta potential and morphology. We chose the smallest resulting NP for loading with DXR because it was more likely to be internalized by cells under the premise of using the EPR effect. Furthermore, we measured the drug-loading amount, encapsulation efficiency and drug release amount of PLV/DXR NPs in media with different pH values. We evaluated cellular uptake of FITC-loaded PLV/DXR NPs to examine the entrance of NPs into tumor cells and the synergism of DXR and LV in combination as well as the cytotoxicity of NPs on MDA-MB-231 (TNBC) and MDA-MB-453 (non-TNBC) cells (Scheme 1).

## Materials and Methods

### Materials

Pullulan (200 kDa) was from Hayashibara (Tokyo). LV and DXR hydrochloride were from J&K Scientific (Beijing). 1-Ethyl-3-(3-dimethylaminopropyl)carbodiimide hydrochloride (EDCI) and 4-dimethylaminopryidine (DMAP) were from Aladdin Reagent (Shanghai). Other reagents such as solvents were from Sinopharm Chemical Reagent Co. (Beijing). The breast cancer cell lines MDA-MB-231 (TNBC) and MDA-MB-453 (non-TNBC) were purchased from the Center for Cell Resource, Shanghai Institutes for Biological Sciences. CellTiterBlue Reagent was from Promega (Madison, WI, USA). Dulbecco’s modified Eagle’s medium (DMEM) (Cat#SH30022.01) was from HyClone (USA), and fetal bovine serum (FBS) (Cat#04-001-1ACS) was from Biological Industries (Israel).

### Synthesis of LV-Grafted Pullulan (PLV)

First, LV succinic acid monoester (LV-SA) was synthesized by the following steps. In brief, 0.60 g succinic anhydride (SA, 6.0 mmol) and 2.0 g LV (5.0 mmol) were dissolved in 20 mL anhydrous pyridine and stirred for 48 h at 50 °C. The resulting reaction solution was slowly poured into a 1500-mL glacial HCl solution of pH 3 under constant stirring to precipitate a large amount of a white precipitate, and the pH of the suspension was adjusted to pH 3 with concentrated HCI, followed by vacuum filtration. The precipitate was then washed with a pH-3 glacial HCl solution, and then with ddH_2_O to neutral to give a crude product. Finally, the crude product was recrystallized from an acetone-water solvent system to obtain pure LV-SA and the yield was 72%. PLV was produced by conjugating the carboxylic acid group of LV-SA with the hydroxyl of pullulan at molar ratios of LV-SA to pullulan of 1/2, 1/3, and 1/4. In brief, 0.5 g (1.03 mmol) prepared LV-SA, 0.15 g DMAP (1.23 mmol) and 0.24 g EDCI (1.23 mmol) were dissolved in 20 mL dimethyl sulfoxide (DMSO) and stirred at 50 °C for 4 h. Pullulan (0.33 g, 1/2; 0.50 g, 1/3; 0.67 g, 1/4) was dissolved in DMSO to obtain a 2% (w/v) solution. Then, the pullulan solution was slowly added to the reaction solution for reaction at 50 °C for 48 h. The reaction mixtures were placed into dialysis bags (MWCO = 8~12 kDa) and dialyzed against 25% ethanol/water and water. Then the samples were freeze-dried [32]. The PLV conjugates with a different feed ratio of LV to pullulan were characterized by FTIR analysis by using an FTIR spectrophotometer (KBr pellets, Nicolet, TM Nexus 470-ESP, Thermo Fisher Scientific, Waltham, MA, USA). PLV conjugates were also confirmed by ^1^H NMR spectroscopy (DMSO-d6 solvent, BRUKER AVANCE-500, Bruker, Billerica, MA, USA), and the degree of substitution (DS) for LV for each conjugate was calculated.

### Preparation and Characterization of NPs

A 50-mg amount of PLV conjugate was dissolved in 12.5 mL DMSO under gentle shaking at 37 °C (to fully swell the conjugate) to obtain 4 mg/mL conjugate solution. A 5-mL amount of conjugate solution was diluted to 2 mg/mL with DMSO for dialysis against 1000 mL distilled water for 8 h with 10 times of exchanges by using a dialysis bag (8~14 kDa). Then, the solution was sonicated by using a probe-type sonifier (GA92-IIDA Ultrasonic cell pulverizer, Suzhou Jiangdong Precision Instruments) at 100 W with pulsing (pulse on 2.0 s, off 2.0 s) for 2 min in an ice-water bath. Self-assembled PLV NPs were obtained and samples were stored at 4 °C. To prepare the PLV/DXR NPs, 2 mg DXR was dissolved in 5 mL DMSO and then added dropwise into 5 mL conjugate solution, then PLV/DXR NPs were prepared by the same method. In a typical procedure for preparing FITC-loaded PLV/DXR NPs, 1 mg DXR was dissolved in 2 mL DMSO and then added dropwise into 2.5 mL conjugate solution. Then, 1 mL FITC solution (0.6 mg/mL) in DMSO was added dropwise, and FITC-loaded PLV/DXR NPs were prepared by the same method.

PLV NPs, PLV/DXR NPs, and FITC-loaded PLV/DXR NPs were filtered through 0.45-μm membranes, then particle sizes, zeta potentials, and polydispersity indexes (PDI) were determined by dynamic light scattering (DLS, Zetasizer 3000 HS, Malvern Instruments, Malvern, UK). The morphologic features of PLV NPs were observed by transmission electron microscopy (TEM; Tecnai G2 20 S-Twin, FEI Hong Kong) at acceleration voltage 80 kV.

### Determination of Loading Capacity and Entrapment Efficiency

A PLV/DXR NP solution (5 mL) was sonicated for 5 min (pulse on 2.0 s, off 2.0 s) to release the drug from NPs. The DXR absorbance in the solution was measured at 480 nm by using an UV-visible spectrophotometer (Shimadzu UV-2550, Kyoto, Japan) to calculate the drug concentrations. DXR encapsulation efficiency (EE) and loading capacity (LC) were calculated as follows:

$$ \mathrm{EE}\%=\frac{\mathrm{amount}\ \mathrm{of}\ \mathrm{drug}\ \mathrm{in}\ \mathrm{NPs}}{\mathrm{amount}\ \mathrm{of}\ \mathrm{total}\ \mathrm{added}\ \mathrm{drug}}\times 100\% $$

$$ \mathrm{LC}\%=\frac{\mathrm{amount}\ \mathrm{of}\ \mathrm{drug}\ \mathrm{in}\ \mathrm{NPs}}{\mathrm{NP}\ \mathrm{weight}}\times 100\% $$

### In Vitro Drug Release

The release profile of DXR from PLV/DXR NPs was measured in 0.1 M phosphate buffer solution at 37 °C by the dialysis method as described [33]. Typically, 6 mL PLV/DXR NPs (equal to 139.4 mg DXR) was transferred into a dialysis bag (MWCO = 3500 Da) and then incubated at 37 °C in 15 mL PBS (pH 7.4 and 5.4). At predetermined times, 3 mL external buffer solution was withdrawn and replaced with 3 mL fresh PBS (pH 7.4 or 5.4). The amount of released DXR was measured by fluorescence spectrophotometry. The percentage rate of drug release (*Q*%) was calculated as follows:

$$ Q\%=\left({C}_n\times V+{V}_n\sum \limits_{t=0}^n{C}_i\right)/\left({W}_{\mathrm{NP}}\times \mathrm{LC}\%\right) $$

where *W* is the NP weight, *C*_*n*_ is the sample concentration at *Tn*, *V* is the total volume of release medium, *V*_*n*_ is the sample volume (2 mL), and *C*_*i*_ is the sample concentration at *T*_*i*_ (*i* = 0, 0.5, 1,…*n* hours, both *V*_*0*_ and *C*_*0*_ are equal to zero).

### Cell Culture

Human breast cancer cell lines MDA-MB-231 and MDA-MB-453 were cultured in DMEM complete medium containing 10% FBS under humid conditions of 37 °C, 5% CO_2_, and normoxia (21% O_2_). The experimental cells were all derived from logarithmic growth-phase cells.

### In Vitro Cytotoxicity

MDA-MB-231 and MDA-MB-453 cells seeded in 96-well plates (4× 10^3^ cells/well in a 100-μL volume) were grown under routine culture conditions for 24 h. Then PLV/DXR NPs with various drug concentrations (mass ratio of DXR and LV was 8.13) were added and incubated for 48 h. Finally, 20 μL CellTiter Blue reagent (Promega) used to measure cell proliferation was added and incubated for 1–4 h at 37 °C. Absorbance at 530/590 nm was measured by using an automatic microplate reader. Cell viability was expressed as a percentage of the absorbance to that for control groups without treatment.

### In Vitro Synergistic Effect Analysis

Isobole analysis was used to quantitatively assess the synergism and antagonism produced by paired drugs. According to Tallarida’s dose-equivalent principle and the Loewe additive model, an isobole is generated, which is a line to define the additive effect of paired drugs [34, 35]. In practice, as we described previously [8], we first acquired the dose-effect curves for free DXR and free LV and after transforming the drug dose and effect, used linear regression to obtain linear regression equations to calculate the combined doses of the paired drugs giving a specified effect. The data for plotting the isobole are illustrated in Table 1. The points on the isobole set the DXR/LV at different ratios to produce a 50% maximum effect. An IC_50_ of the paired drug dose located below the isobole indicates a synergistic effect, whereas an IC_50_ above the isobole indicates an antagonistic effect.

**Table 1:** Data for plotting the isobole

DXR		LV		Additive effect, (*P*_*d*_)_*D*_ + (*P*_*d*_)_*L*_	Addition dose, (Dose_*D*_ + Dose_*L*_) (μM)	Dose pair, (Dose_*D*_,Dose_*L*_) (μM)
*P*_*d*_ = 0.4865 − 0.2606 lgdose (*R*^2^ = 0.9529)		*P*_*d*_ = 1.092 − 0.05881 dose (*R*^2^ = 0.9808)				
(1-*P*_*d*_) C	Dose_*D*_ (μM)	(1-*P*_*d*_)_*L*_	Dose_*L*_ (μM)			
0%	0	50%	10.066	50%	10.066	(0,10.066)
10%	0.0259	40%	8.3659	50%	8.3918	(0.0259,8.3659)
20%	0.0627	30%	6.6655	50%	6.7282	(0.0627,6.6655)
30%	0.1516	20%	4.9651	50%	5.1167	(0.1516,4.9651)
40%	0.3668	10%	3.2648	50%	3.6316	(0.3668,3.2648)
50%	0.8876	0%	0	50%	0.8876	(0.8876,0)

Where “*P*_*d*_” is cell viability and (1-*P*_*d*_) is the cell death

### In Vitro Cellular Uptake

PLV/DXR FITC NPs were obtained by dialysis. The cellular uptake of PLV/DXR FITC NPs was tested by using a fluorescence microplate reader. MDA-MB-231 and MDA-MB-453 cells were seeded (2 × 10^5^/well) on 2.5-cm dishes and incubated at 37 °C for 24 h. Cells were then incubated with concentrations of PLV-DXR FITC NPs at 37 °C for 1, 2, and 4 h. The culture medium was then removed and washed twice with cold PBS. Cells were fixed with 4% paraformaldehyde for 5 min and mounted on slides by using mounting medium containing DAPI. Then, the cellular uptake of NPs was visualized by fluorescence microscopy.

### Statistical Analysis

Data are expressed as mean ± SD and were analyzed by Student’s *t* test. Differences were considered statistically significant at *P* < 0.05.

## Results

### In Vitro Cytotoxicity and Synergistic Effect of DXR and LV

**Fig. 1:**
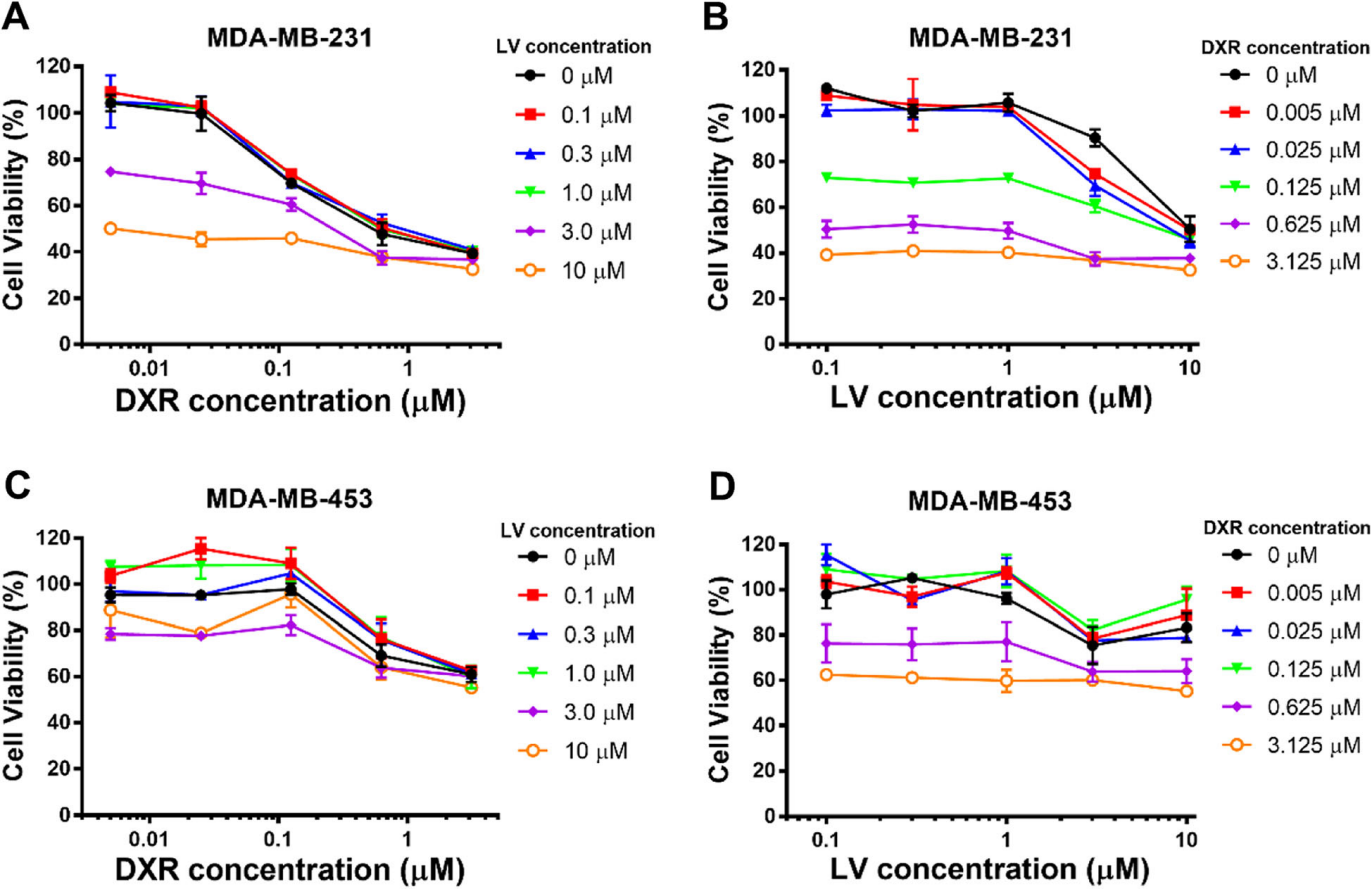
Cytotoxicity of free doxorubicin (DXR) and free lovastatin (LV) in breast cancer cells. In vitro cytotoxicity of free DXR, LV, and DXR and LV combined against MDA-MB-231 (**a**, **b**) and MDA-MB-453 (**c**, **d**) cancer cells

To evaluate the inhibitory effect of LV and DXR against TNBC and non-TNBC cell proliferation, cell viability was assessed by Alamar blue assay. We first determined the inhibitory effect of LV and DXR on the two TNBC cell lines by a free drug test. A series of LV/DXR concentration ratios were obtained by setting the concentration of one drug constant and changing that of the other drug. For the MDA-MB-231 cell line, both LV and DXR conferred a concentration-dependent inhibition, but LV had a significant inhibitory effect at concentrations up to 3.0 μM (Fig. 1).

**Fig. 2:**
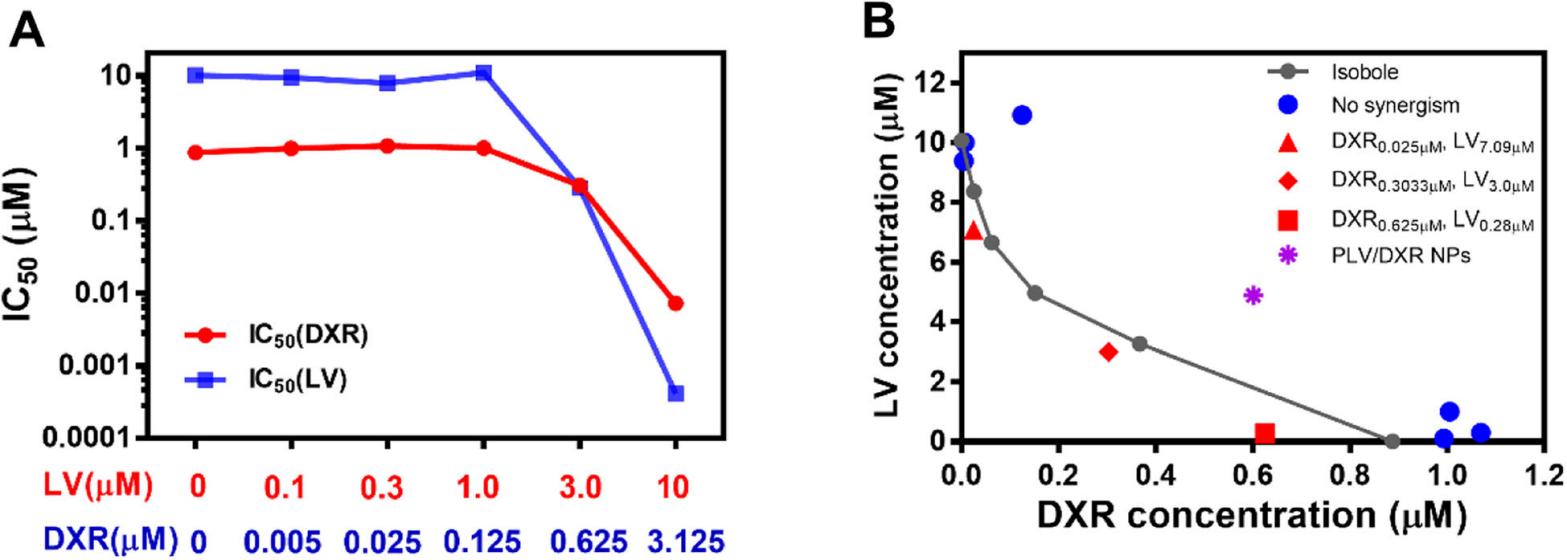
Synergism of DXR and LV. IC_50_ values for DXR and LV with different LV and DXR concentrations calculated by GraphPad Prism 7 (**a**) and the isobole method (**b**)

The IC_50_ values for LV under different DXR treatments and for DXR under different LV treatments are shown in Table 2, and we also plotted these IC_50_ values (Fig. 2a) to visually reflect the combined effect of DXR/LV. The IC_50_ for free DXR alone was 0.865 μM, and that for free LV alone was 10.07 μM. When the concentration of DXR increased from 0.125 to 0.625 μM (fivefold increase), the IC_50_ for LV decreased from 10.92 to 0.28 μM (39-fold reduction), and when the DXR concentration increased from 0.625 to 3.125 μM (fivefold increase), the IC_50_ value for LV decreased from 0.28 to 0.0004085 μM (685-fold reduction). Similarly, when the LV concentration increased from 1.0 to 3.0 μM (threefold increase), the IC_50_ value for DXR decreased from 1.005 to 0.3033 μM (3.3-fold reduction) and when the LV concentration increased from 3.0 to 10 μM (3.3-fold increase), the IC_50_ value for DXR decreased from 0.3033 to 0.007196 μM (42-fold reduction). Thus, for the same inhibition rate of MDA-MD-231 cell proliferation, DXR could significantly reduce the amount of LV needed, and LV could also significantly reduce the amount of DXR needed. This finding is essential for tumor chemotherapy because DXR is a highly potent chemotherapeutic drug with undesirable adverse side effects and should be kept in low concentration, and LV has minimal adverse side effects [36]. For MDA-MB-453 cells, DXR showed a concentration-dependent inhibition, but LV showed no significant inhibition at the concentrations examined. In addition, DXR had a significant inhibitory effect on MDA-MB-453 cells at concentrations up to 3.0 μM, which was far less effective than for MDA-MB-231 cells (Fig. 1). We recently found that LV selectively inhibited a panel of TNBC cell lines as compared with non-TNBC cell lines [8]; therefore, the combination of LV and DXR could be suitable for treating TNBC but not non-TNBC cells.

**Table 2:** DXR/LV in combination achieving 50% inhibitory

DXR (μM)	LV (μM)	LV (μM)	DXR (μM)
0	10.07	0	0.865
0.005	9.387	0.1	0.993
0.025	7.090	0.3	1.069
0.125	10.92	1.0	1.005
0.625	0.28	3.0	0.3033
3.125	0.0004085	10.0	0.007196

To further quantitatively assess the synergism of DXR and LV, we used the isobole method. With different DXR/LV ratios to achieve 50% maximal effect (Table 1), we plotted an isobole that indicated the additive effect of the DXR/LV ratio (Fig. 2b). The IC_50_ doses of DXR/LV at 0.025/7.09, 0.3033/3.0, and 0.625/0.28 (μM/μM) were below the isobole, which suggests that DXR/LV at these ratios would produce a synergistic effect. Thus, this synergistic effect (1 + 1 > 2) would further achieve pharmacologically statistically minimized drug doses with maximized drug efficacy [37]. These three synergistic ratios appeared to represent these results: (1) a low dose of DXR (0.025 μM) could better enhance the therapeutic efficacy of LV because 0.025 μM DXR could produce a larger negative slope for the dose-effect curve of LV (Fig. 2b); (2) with high DXR concentration, adding a small amount of LV could greatly assist the DXR effect against MDA-MB-231 cells because the IC_50_ for DXR alone was 0.865 μM, but adding only 0.28 μM LV reduced the IC_50_ of DXR to 0.625 μM, that is, 0.28 μM LV completely replaced the efficacy of 0.24 μM DXR (Table 2); and (3) when the dose of LV was about 10 times that of DXR, the DXR/LV ratio should have the best synergistic effect because the point (0.3033, 3.0) was farthest from the isobole (Fig. 2b).

### Synthesis and Structural Characterization of PLV Conjugates

**Scheme 2:**
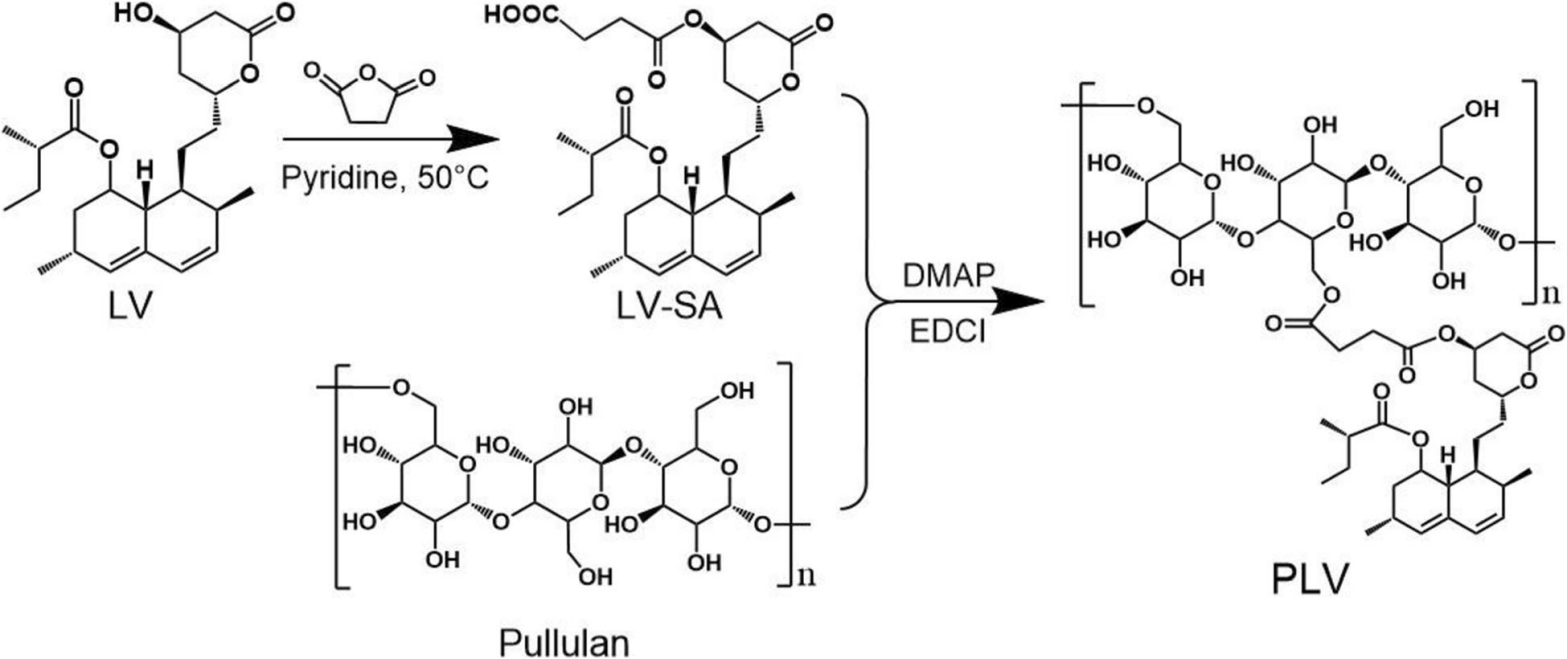
Chemical synthesis of amphiphilic PLV conjugates. LV and succinic anhydride (SA) reacted under the catalysis of pyridine to form LV-SA at 50 °L. Then LV-SA was linked to pullulan by an ester bond under the catalysis of EDC and DMAP to form the PLV polymer

**Fig. 3:**
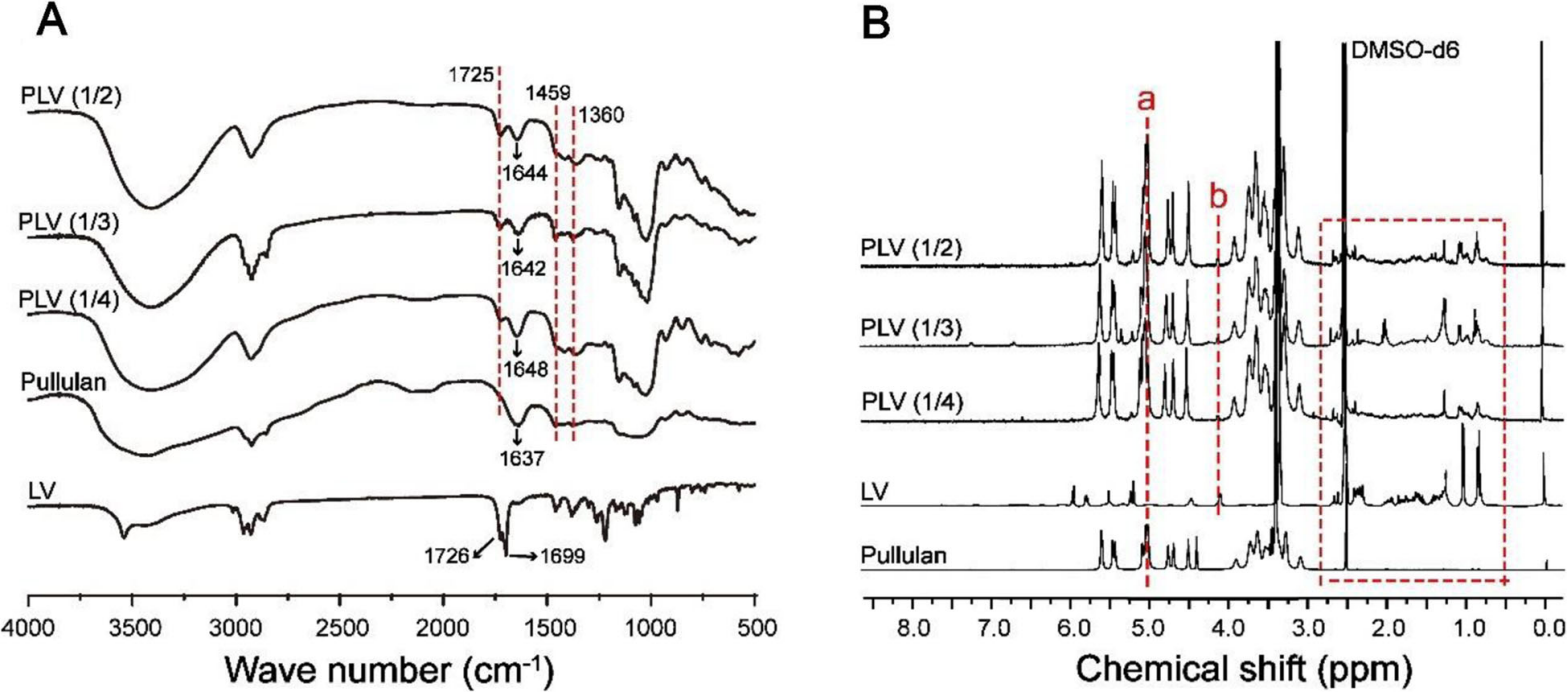
Structural characterization of pullulan-lovastatin (PLV) conjugates. **a** FTIR spectra for pullulan, LV, PLV (1/2), PLV (1/3), and PLV (1/4), peaking at 1644, 1642, and 1648 cm^−1^ as characteristic peaks of pullulan. The dotted lines at 1360, 1459, and 1725 cm^−1^ show the bending vibration peaks of –CH_3_– and –CH_2_– for LV. **b** ^1^H NMR spectra for pullulan, LV, PLV (1/2), PLV (1/3), and PLV (1/4)

The amphiphilic PLV conjugates were synthesized as shown in Scheme 2. PLV was obtained by esterification of pullulan and LV. The structures of PLV conjugates were confirmed by FTIR and ^1^H NMR spectra (Fig. 3). According to FTIR analysis (Fig. 3a), PLV conjugates were successfully produced. For the spectra of PLV conjugates, in addition to maintaining the characteristic peaks of pullulan (1644, 1642, and 1648 cm^−1^), the enhanced peaks at 1459 cm^−1^ and 1360 cm^−1^ also showed the bending vibration peaks of –CH_3_ and –CH_2_–, respectively, for LV. Furthermore, we observed high wave number shift of –C=O stretching absorption peaks (ester bonding in LV) at 1725 cm^−1^ from PLV conjugates because of the newly generated ester bond, and these peaks were enhanced with an increased molar ratio of LV to pullulan (Fig. 3a).

We confirmed the successful synthesis of PLV conjugates by ^1^H NMR spectra (Fig. 3b). The ^1^H-NMR spectra showed the characteristic peaks of pullulan and new peaks assigned to the CH_3_, CH_2_, and CH protons of the LV moiety at 0.5~2.7 ppm (red frame), which indicated the successful conjugation of LV to pullulan. We used the characteristic peaks of pullulan at 4.94~5.14 ppm (a) corresponding to the α-1,6 and α-1,4 glycosidic bond protons of C_1_ to define the glucose units in pullulan [38]. The signals at 4.12 ppm (b) corresponded to the proton of the C_13_ in LV. Thus, the DS of LV residues per 100 glucose units of pullulan was calculated by the ratio of C_13_ protons (4.05~4.15 ppm) of LV to sugar protons (4.94~5.14 ppm) with the following equation: DS = *I*_4.05~4.15_/*I*_4.94~5.14_ × 100%. The DS data for LV are in Table 3.

**Table 3:** Characterizations of PLV nanoparticles

Sample	DS	LC_*L*_%	LC_*D*_%	EE%	Zeta potential (mV)	Average size (nm)	PDI
PLV NP (1/2)	7.87%	15.99%	–	–	− 11.66	177.2	0.138
PLV NP (1/3)	3.58%	8.09%	–	–	− 10.51	189.7	0.160
PLV NP (1/4)	3.06%	7.01%	–	–	− 8.30	219.8	0.050
PLV/DXR NP	7.87%	15.69%	1.93%	20.92%	− 10.51	225.6	0.073
FITC-loaded PLV/DXR NP	7.87%	–	–	–	− 15.09	253.8	0.078

### Characterizations of PLV NPs

**Fig. 4:**
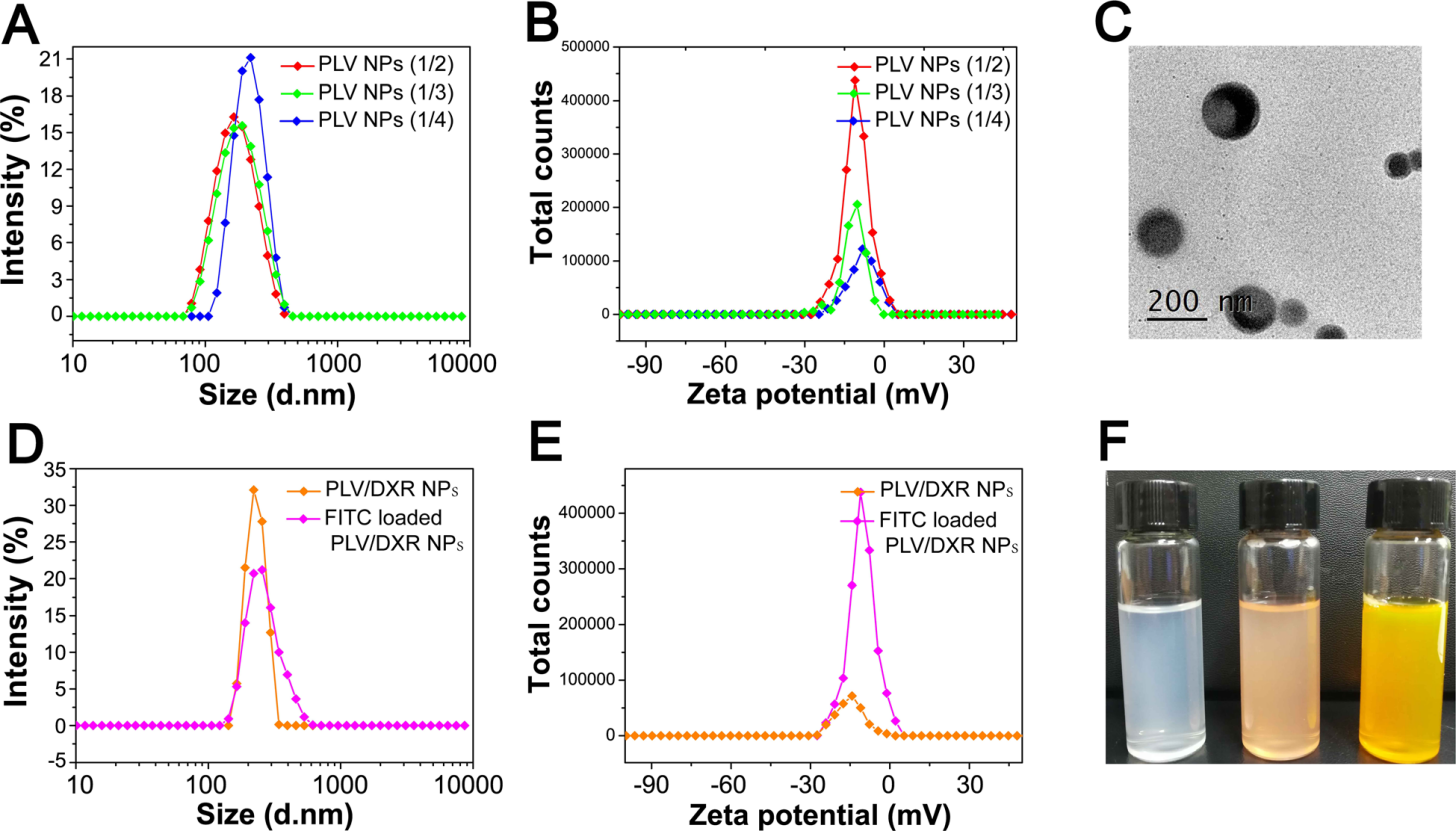
Characterization of nanoparticles (NPs). **a** Particle size and distribution of PLV. **b** Zeta potential of PLV. c Transmission electron microscopy image of PLV (1/2). **d** Particle size and distribution of PLV/DXR and FITC-loaded PLV/DXR. **e** Zeta potential of PLV/DXR and FITC-loaded PLV/DXR. **f** Photograph of PLV, PLV/DXR, and FITC-loaded PLV/DXR

The synthesized PLV conjugate could self-assemble into micelles in an aqueous solution. The average size and polydispersity index (PDI) were 177.2 nm and 0.138, respectively, for PLV (1/2) NPs; 189.7 nm and 0.160 for PLV (1/3) NPs; and 219.8 nm and 0.050 for PLV (1/4) NPs (Table 3; Fig. 4a, b). In addition, the zeta potential for PLV (1/2), PLV (1/3), and PLV (1/4) NPs was − 11.66, − 10.51, and − 8.30 mV, respectively. The size of PLV NPs decreased with increasing DS of LV, with no significant change in zeta potential. The change in size is mainly attributed to the strengthening of hydrophobic interaction among LV groups, which results in the formation of more compact hydrophobic cores [39]. We previously studied pullulan NPs with different DS values of cholesterol and found that to a certain extent, the greater the hydrophobic DS of the amphiphilic polymer, the smaller the size of the formed NPs [33]. In this study, because the DS of the hydrophobic group LV was larger, the size of the NPs was also smaller. Also, TEM images (Fig. 4c) showed that the NPs were generally in a spherical shape with good monodispersity. PLV (1/2) NPs, with the highest DS leading to the highest LV loading and the smallest size, was selected for the following experiments.

PLV/DXR NPs also exhibited spherical morphology and a larger size, 225.6 nm, than empty PLV NPs. The encapsulation efficiency and loading capacity of PLV/DXR NPs was 20.92% and 1.93%, respectively. The hydrophobic interaction between the hydrophobic core of the NPs and the hydrophobic group of the drug determines the amount of drug loading in the NPs. Considering that the water solubility of DXR hydrochloride used in this experiment was slightly larger, the hydrophobic interaction between DXR and the hydrophobic core of the NPs was weak, which led to a lesser amount of DXR actually encapsulated in the NPs. To further clarify the uptake of NPs by tumor cells, FITC was loaded into PLV/DXR NPs. With FITC loading, the mean size of FITC-loaded PLV/DXR NPs was 253.8 nm and mean zeta potential − 15.09 mV (Fig. 4d, e).

### In Vitro Drug Release

**Fig. 5:**
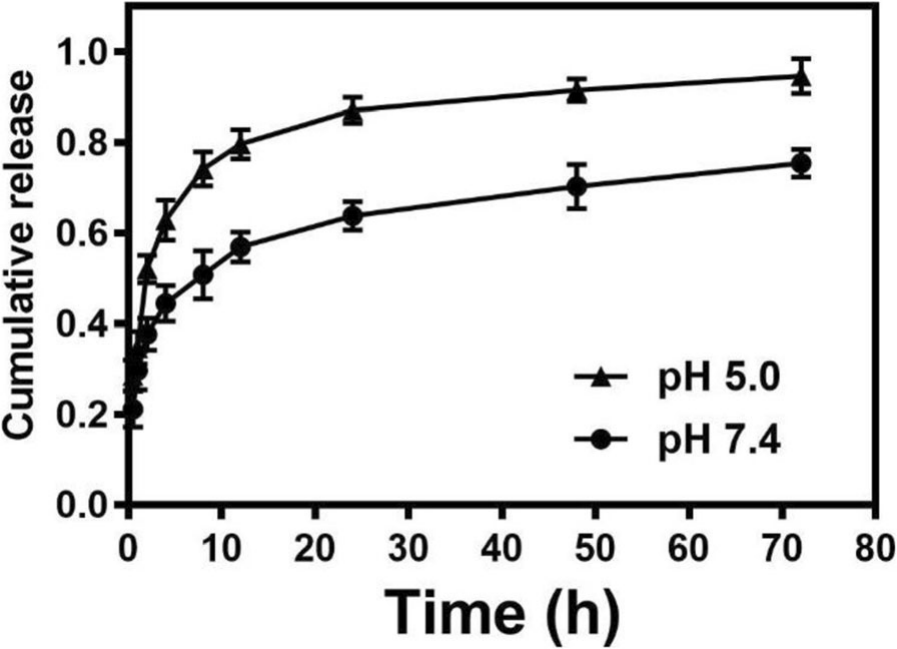
In vitro release profiles of DXR from PLV/DXR in phosphate buffered saline at pH 7.4 vs 5.4 at different times after dialysis

To reveal the release behavior of DXR from PLV/DXR NPs, in vitro DXR release profiles were assessed in PBS at pH 7.4 and 5.4, mimicking the conditions in normal physiological tissues and the intracellular microenvironment, respectively. PLV/DXR NPs exhibited two phases of DXR release: a rapid release in the first 8 h and a sustained release thereafter (Fig. 5), which is consistent with the release profile of typical NPs. In addition, with a decrease of pH from 7.4 to 5.4, the cumulative DXR release was accelerated significantly up to 76.15% and 97.90%, respectively, at 72 h. Therefore, the release of DXR from PLV/DXR NPs was pH-sensitive to some extent, which is especially useful in enhancing the therapeutic efficacy and reducing side effects in vivo [40].

### Cellular Uptake of FITC-Loaded PLV/DXR NPs

**Fig. 6:**
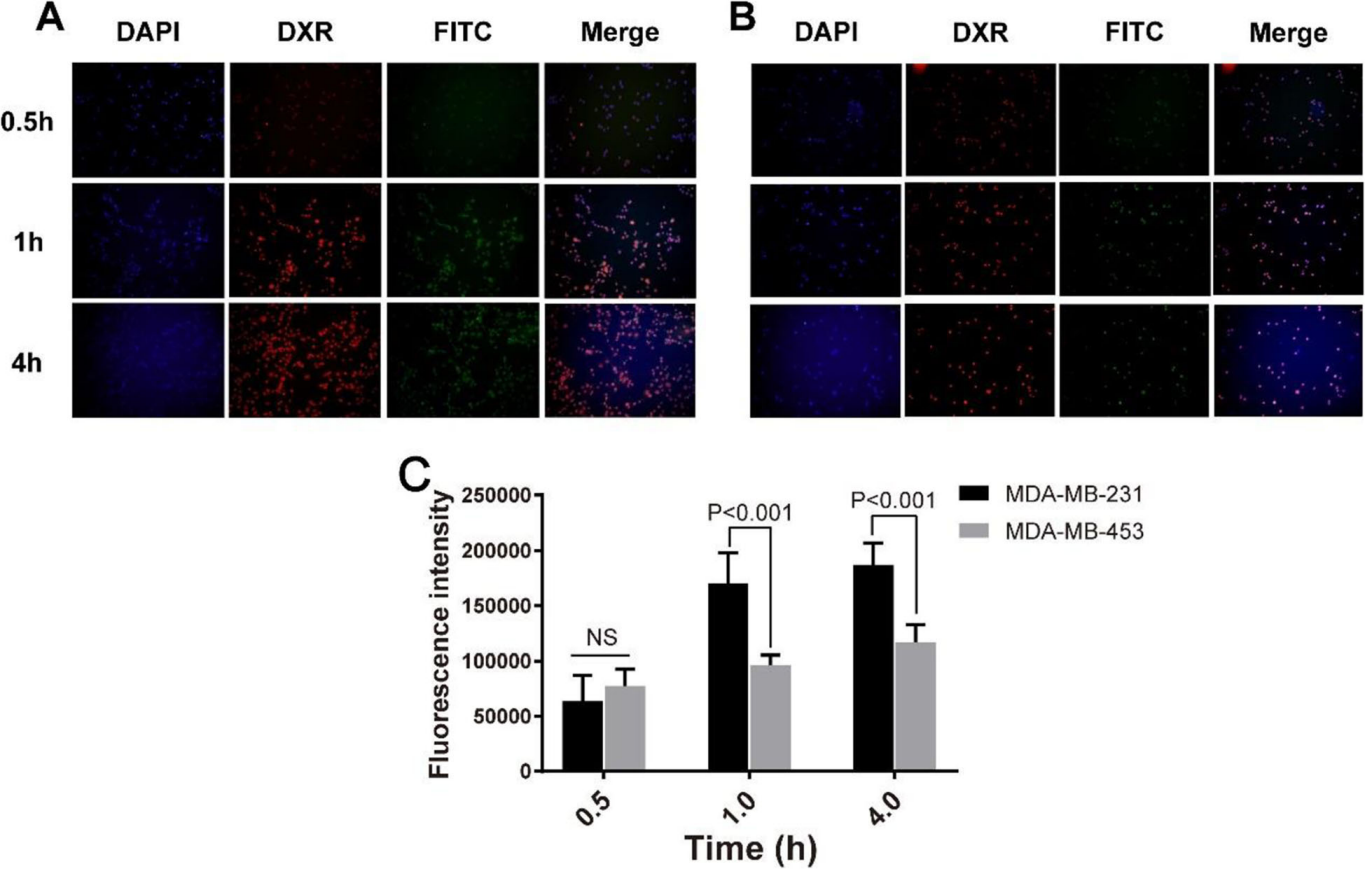
Breast cancer cell uptake of drugs loaded in PLV/DXR NPs. Fluorescence microscopy images of FITC-loaded PLV/DXR taken up by MDA-MB-231 (**a**) and MDA-MB-453 (**b**) cells at 0.5, 1, and 4 h (blue for DAPI, red for DXR, green for FITC). **c** Quantitative analysis of cellular uptake of DXR in MDA-MB-231 and MDA-MB-453 cells after PLV/DXR treatment

We obtained fluorescence microscopy images of MDA-MB-231 (Fig. 6a) and MDA-MB-453 (Fig. 6b) cells at 0.5, 1, and 4 h, respectively, after exposure to FITC-loaded PLV/DXR NPs and found a time-dependent increase in intensity of both red and green fluorescence from 0.5 to 4 h after NP treatment (Fig. 6), which indicates a time-dependent cellular uptake of DXR and FITC-loaded NPs, respectively. Also, the amount of FITC entering MDA-MB-231 cells was greater than that entering MDA-MB-453 cells. Further quantification of the fluorescence intensity revealed a significant difference in uptake of DXR between MDA-MB-231 and MDA-MB-453 cells at 1 h after NP treatment (Fig. 6c).

### In Vitro Cytotoxicity of PLV/DXR NPs

**Fig. 7:**
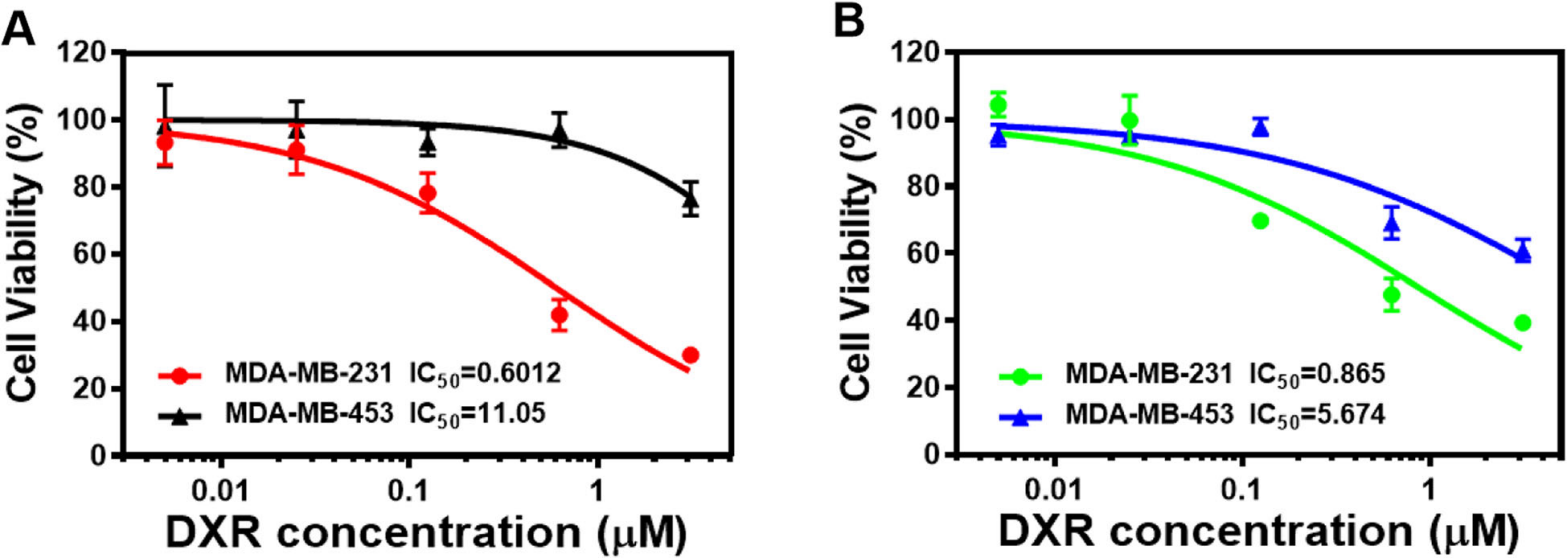
In vitro cytotoxicity of NP-delivered drug vs free drug. Cytotoxicity of PLV/DXR (**a**) and free DXR (**b**) against MDA-MB-231 and MDA-MB-453 cells

After confirming the inhibitory effect of DXR and LV on the viability of MDA-MB-231 and MDA-MB-453 cells and demonstrating the synergistic effect of DXR and LV, we determined the in vitro cytotoxicity of PLV/DXR NPs on these two cell lines. MDA-MB-231 and MDA-MB-453 cells were incubated with PLV/DXR NPs at a concentration equivalent to that of the free DXR. The cytotoxicity of PLV/DXR NPs increased with increasing DXR concentration and was greater for MDA-MB-231 than MDA-MB-453 cells (Fig. 7a). This finding is consistent with the cytotoxicity trend of free drugs (Fig. 7b) and indicates that loading free drugs in NPs did not affect the inhibitory behavior of the drugs. PLV/DXR NPs had higher inhibitory effect on the proliferation of MDA-MB-231 than MDA-MB-453 cells, which was consistent with the preferential inhibitory effect of LV on TNBC. Growth inhibition of MDA-MB-231 cells was greater with PLV/DXR NPs than free DXR (IC_50_ 0.6012 vs 0.865 μM), which suggests that NPs might facilitate cellular uptake and retention of the loaded drug inside the cell, causing enhanced cytotoxicity as compared with free drug.

## Discussion

In this study, PLV/DXR NPs prepared for in vitro drug-release profiling sustainably released DXR within 72 h, which was robust at pH 5.4 (97.9%). PLV/DXR NPs more strongly inhibited proliferation of TNBC MDA-MB-231 than non-TNBC MDA-MB-453 cells. Both cell lines showed time-dependent uptake of the NPs, but MDA-MB-231 cells took up more NPs than did MDA-MB-453 cells. Thus, pullulan-based NP co-delivery of LV and DXR could efficiently inhibit TNBC breast cancer cell proliferation, which may suggest a powerful drug delivery system for treating TNBC.

Recently, the use of LVs in TNBC prevention and treatment has gained recognition, and studies have further shown that LV preferentially inhibits the proliferation of TNBC cells and induces apoptosis [10, 41]. However, clinically, LVs are mostly in oral preparations, but other anti-tumor drugs for chemotherapy including DXR are mostly in injection form. This situation results in undesirable chemotherapeutic efficacy for combining LV and other drugs, for difficulty in reaching the tumor tissue at the expected dose and time. The formulation of LV as an injection is difficult to achieve because of its water insolubility. Therefore, nano delivery systems are necessary for LV to be used for anti-tumor efficacy and also allow the co-encapsulation of several drugs for easier combination chemotherapy.

Our group recently prepared a cerasome-based nanocomposite to encapsulate LV, which solved the issue of the water solubility of LV to some extent, but the drug-loading efficiency was in general not high (5~6%) [8]. Zhang et al. developed camptothecin-floxuridine conjugate nanocapsules to load LV. These nanocapsules, consisting of two US Food and Drug Administration-approved chemotherapeutic drugs, achieved ultra-high drug loading capacity for camptothecin-floxuridine (68.3%), but the loading efficiency for LV was still low (2.8%) [29]. In the present study, we used LV for the hydrophobic modification of pullulan to obtain amphiphilic PLV conjugates and then prepared PLV/DXR NPs, which not only solved the issue of water solubility of LV, but also yielded high drug-loading capacity (15.69% for LV and 1.93% for DXR).

The strategy of encapsulating two or more anti-tumor drugs into a nanocarrier has been widely accepted for efficient drug delivery [42]. Sui et al. used DXR-grafted-pullulan NPs loading sorafenib to achieve synergistic chemotherapy against murine breast carcinoma [30]. Our study used a similar methodology for PLV/DXR NPs, displaying higher inhibitory efficacy for MDA-MB-231 cells as compared with DXR alone. However, PLV/DXR NPs did not show synergistic effects, which might be related to the lag of NP endocytosis and LV release, which deserves further study.

The combination index has been widely used to quantify the synergistic effects of two drugs [43–45]. The isobologram analysis method used in quantitative assessment of synergistic effects can effectively avoid false-positive results [35]. In this study, we screened the optimal synergy ratio from a range of DXR/LV ratios based on isobologram analysis.

Our research provides a rationale for the design of a more efficient co-delivery system to elicit synergistically enhanced inhibitory effects on TNBC. Since our work is preliminary and based on the premise that EPR effect is feasible, further investigations of the in vivo effectiveness of this novel co-delivery system on TNBC using orthotopic breast tumor growth models and patient-derived xenograft models are warranted.

## Conclusion

In this study, we developed novel dual drug-loaded NPs by chemically grafting LV to pullulan and physically encapsulating DXR, which had excellent monodispersity, high drug loading capacity, and good drug release behavior. The PLV/DXR NPs showed sustainable pH-sensitive release behavior of DXR. PLV/DXR NPs more effectively internalized and inhibited proliferation of TNBC MDA-MB-231 than non-TNBC MDA-MB-453 cells. In addition, we demonstrated the synergistic effect of a free DXR/LV mixture, which provides a combination chemotherapy regiment for potential future clinical treatment of TNBC.

## Data Availability

The conclusions made in this manuscript are based on the data which are all presented and shown in this paper.

## Abbreviations

^1^H NMR: Proton nuclear magnetic resonance
DLS: Dynamic light scattering
DMAP: 4-Dimethylaminopryidine
DMEM: Dulbecco’s modified Eagle’s medium
DMSO: Dimethyl sulfoxide
DS: Degrees of substitution
DXR: Doxorubicin
EDCI: 1-Ethyl-3-(3-dimethylaminopropyl)carbodiimide hydrochloride
EE: Encapsulation efficiency
EPR: Enhanced permeability and retention
ER: Estrogen receptor
FBS: Fetal bovine serum
FTIR: Fourier transform infrared
HER2: Human epidermal growth factor receptor 2
LC: Loading capacity
LV: Lovastatin
LV-SA: Lovastatin succinic acid monoester
NP: Nanoparticle
PDI: Polydispersity indexes
PLV: Pullulan-encapsulated lovastatin
PLV/DXR NPs: Doxorubicin-loaded PLV nanoparticles
PR: Progesterone receptor
SA: Succinic anhydride
TNBC: Triple-negative breast cancer

## References

[1] Nofech-Mozes S, Trudeau M, Kahn HK (2009). Patterns of recurrence in the basal and non-basal subtypes of triple-negative breast cancers. Breast Cancer Res. Treat..

[2] Lee A, Djamgoz MBA (2018). Triple negative breast cancer: emerging therapeutic modalities and novel combination therapies. Cancer Treat. Rev..

[3] Bianchini G, Balko JM, Mayer IA (2016). Triple-negative breast cancer: challenges and opportunities of a heterogeneous disease. Nat Rev Clin Oncol.

[4] Acharya S, Sahoo SK (2011). PLGA nanoparticles containing various anticancer agents and tumour delivery by EPR effect. Adv. Drug Deliv. Rev..

[5] Maeda H, Matsumura Y (2011). EPR effect based drug design and clinical outlook for enhanced cancer chemotherapy. Adv Drug Deliv Rev..

[6] Torchilin V (2011). Tumor delivery of macromolecular drugs based on the EPR effect. Adv Drug Deliv Rev..

[7] Tobert JA (2003). Lovastatin and beyond: the history of the HMG-CoA reductase inhibitors. Nat Rev Drug Discov.

[8] Song L, Tao X, Lin L (2018). Cerasomal lovastatin nanohybrids for efficient inhibition of triple-negative breast cancer stem cells to improve therapeutic efficacy. ACS Appl Mater Interfaces.

[9] Yang T, Yao H, He G (2016). Effects of lovastatin on MDA-MB-231 breast cancer cells: an antibody microarray analysis. J Cancer.

[10] Campbell MJ, Esserman LJ, Zhou Y (2006). Breast cancer growth prevention by statins. Cancer Res..

[11] Wolfe AR, Debeb BG, Lacerda L (2015). Simvastatin prevents triple-negative breast cancer metastasis in pre-clinical models through regulation of FOXO3a. Breast Cancer Res. Treat..

[12] Fujii T, Le Du F, Xiao L (2015). Effectiveness of an adjuvant chemotherapy regimen for early-stage breast cancer: a systematic review and network meta-analysis. JAMA Oncol.

[13] Feleszko W, Zagozdzon R, Golab J (1998). Potentiated antitumour effects of cisplatin and lovastatin against MmB16 melanoma in mice. Eur J Cancer.

[14] Holstein SA, Hohl RJ (2001). Interaction of cytosine arabinoside and lovastatin in human leukemia cells. Leuk Res.

[15] Martirosyan A, Clendening JW, Goard CA (2010). Lovastatin induces apoptosis of ovarian cancer cells and synergizes with doxorubicin: potential therapeutic relevance. BMC Cancer.

[16] Wang E, Casciano CN, Clement RP (2001). HMG-CoA reductase inhibitors (statins) characterized as direct inhibitors of P-glycoprotein. Pharm Res.

[17] Goard CA, Mather RG, Vinepal B (2010). Differential interactions between statins and P-glycoprotein: implications for exploiting statins as anticancer agents. Int J Cancer.

[18] Chaisit T, Siripong P, Jianmongkol S (2017). Rhinacanthin-C enhances doxorubicin cytotoxicity via inhibiting the functions of P-glycoprotein and MRP2 in breast cancer cells. Eur J Pharmacol..

[19] Cai L, Xu G, Shi C (2015). Telodendrimer nanocarrier for co-delivery of paclitaxel and cisplatin: a synergistic combination nanotherapy for ovarian cancer treatment. Biomaterials.

[20] Miao L, Guo S, Zhang J (2014). Nanoparticles with precise ratiometric co-loading and co-delivery of gemcitabine monophosphate and cisplatin for treatment of bladder cancer. Adv Funct Mater.

[21] Cheetham AG, Zhang P, Lin YA (2014). Synthesis and self-assembly of a mikto-arm star dual drug amphiphile containing both paclitaxel and camptothecin. J Mater Chem B.

[22] Ganta S, Amiji M (2009). Coadministration of paclitaxel and curcumin in nanoemulsion formulations to overcome multidrug resistance in tumor cells. Mol Pharm.

[23] Lehar J, Krueger AS, Avery W (2009). Synergistic drug combinations tend to improve therapeutically relevant selectivity. Nat Biotechnol.

[24] Aryal S, Hu CJ, Zhang L (2010). Combinatorial drug conjugation enables nanoparticle dual-drug delivery. Small.

[25] Fan L, Li F, Zhang H (2010). Co-delivery of PDTC and doxorubicin by multifunctional micellar nanoparticles to achieve active targeted drug delivery and overcome multidrug resistance. Biomaterials.

[26] Duan X, Xiao J, Yin Q (2013). Smart pH-sensitive and temporal-controlled polymeric micelles for effective combination therapy of doxorubicin and disulfiram. ACS Nano.

[27] Feng C, Zhang H, Chen J (2019). Ratiometric co-encapsulation and co-delivery of doxorubicin and paclitaxel by tumor-targeted lipodisks for combination therapy of breast cancer. Int J Pharm.

[28] Guo S, Lin CM, Xu Z (2014). Co-delivery of cisplatin and rapamycin for enhanced anticancer therapy through synergistic effects and microenvironment modulation. ACS Nano.

[29] Zhang N, Liang X, Gao C (2018). Loading lovastatin into camptothecin-floxuridine conjugate nanocapsules for enhancing anti-metastatic efficacy of cocktail chemotherapy on triple-negative breast cancer. ACS Appl Mater Interfaces.

[30] Sui J, Cui Y, Cai H (2017). Synergistic chemotherapeutic effect of sorafenib-loaded pullulan-Dox conjugate nanoparticles against murine breast carcinoma. Nanoscale.

[31] Tao X, Jin S, Wu D (2015). Effects of particle hydrophobicity, surface charge, media pH value and complexation with human serum albumin on drug release behavior of mitoxantrone-loaded pullulan nanoparticles. Nanomaterials (Basel).

[32] Lv Y, Xu C, Zhao X (2018). Nanoplatform assembled from a CD44-targeted prodrug and smart liposomes for dual targeting of tumor microenvironment and cancer cells. ACS Nano.

[33] Tao X, Xie Y, Zhang Q (2016). Cholesterol-modified amino-pullulan nanoparticles as a drug carrier: comparative study of cholesterol-modified carboxyethyl pullulan and pullulan nanoparticles. Nanomaterials.

[34] Tallarida RJ (2012). Revisiting the isobole and related quantitative methods for assessing drug synergism. J Pharmacol Exp Ther..

[35] Tallarida RJ (2011). Quantitative methods for assessing drug synergism. Genes Cancer.

[36] Gupta SK, Garg A, Bär C (2018). Quaking inhibits doxorubicin-mediated cardiotoxicity through regulation of cardiac circular RNA expression. Circulation research.

[37] Mathur M, Devi Vemula K (2018). Investigation of different types of nano drug delivery systems of atorvastatin for the treatment of hyperlipidemia. Drug Dev Ind Pharm.

[38] Akiyoshi K, Deguchi S, Moriguchi N (1993). Self-aggregates of hydrophobized polysaccharides in water. Formation and characteristics of nanoparticles. Macromolecules.

[39] Dinh HTT, Tran PHL, Duan W (2017). Nano-sized solid dispersions based on hydrophobic-hydrophilic conjugates for dissolution enhancement of poorly water-soluble drugs. Int J Pharm.

[40] Liu J, Huang Y, Kumar A (2014). pH-sensitive nano-systems for drug delivery in cancer therapy. Biotechnol Adv.

[41] Dimitroulakos J, Ye LY, Benzaquen M (2001). Differential sensitivity of various pediatric cancers and squamous cell carcinomas to lovastatin-induced apoptosis: therapeutic implications. Clin Cancer Res.

[42] Gao C, Bhattarai P, Chen M (2018). Amphiphilic Drug Conjugates as Nanomedicines for Combined Cancer Therapy. Bioconjug Chem.

[43] Li Y, Lin J, Ma J (2017). Methotrexate-camptothecin prodrug nanoassemblies as a versatile nanoplatform for biomodal imaging-guided self-active targeted and synergistic chemotherapy. ACS Appl Mater Interfaces.

[44] Mielczarek L, Krug P, Mazur M (2019). In the triple-negative breast cancer MDA-MB-231 cell line, sulforaphane enhances the intracellular accumulation and anticancer action of doxorubicin encapsulated in liposomes. Int J Pharm.

[45] Xiao B, Si X, Han MK (2015). Co-delivery of camptothecin and curcumin by cationic polymeric nanoparticles for synergistic colon cancer combination chemotherapy. J Mater Chem B.

